# Analysis of factors related to cognitive impairment in a community‐based, complete enumeration survey in Japan: the Nakayama study

**DOI:** 10.1111/psyg.13012

**Published:** 2023-07-22

**Authors:** Taku Yoshida, Takaaki Mori, Hideaki Shimizu, Ayumi Tachibana, Yuta Yoshino, Shinichiro Ochi, Kiyohiro Yamazaki, Yuki Ozaki, Kentaro Kawabe, Fumie Horiuchi, Kenjiro Komori, Jun‐ichi Iga, Shu‐ichi Ueno

**Affiliations:** ^1^ Department of Psychiatry Zaidan Niihama Hospital Niihama Japan; ^2^ Department of Neuropsychiatry Ehime University Graduate School of Medicine Toon Japan; ^3^ Department of Psychiatry Heisei Hospital Ozu Japan; ^4^ Department of Psychiatry Matsukaze Hospital Shikokuchuou Japan; ^5^ Office of Psychology, Department of Psychiatry Juzen‐Yurinoki Hospital Niihama Japan

**Keywords:** Alzheimer's disease, cognitive impairment's factors, complete enumeration survey, diabetes mellitus

## Abstract

**Background:**

The number of patients with cognitive disorders is rapidly increasing in the world, becoming not only a medical problem, but also a social problem. There have been many reports that various factors are associated with cognitive dysfunction, but the factors have not yet been fully identified. This was a community‐based complete enumeration study which aimed to identify risk and protective factors for dementia.

**Methods:**

The first phase included all residents aged 65 years or older in a town in Japan. They completed many examinations, such as living conditions questionnaires, physical examination, Mini‐Mental State Examination, and brain magnetic resonance imaging. The participants with suspected cognitive impairment underwent additional examinations for detailed evaluation in the second phase. Statistical analysis was performed to identify risk and protective factors for dementia after all participants were diagnosed.

**Results:**

There were 927 participants in the baseline evaluation; 611 (65.9%) were healthy, 165 (17.8%) had mild cognitive impairment (MCI), and 151 (16.3%) had dementia. The age‐standardised prevalence of dementia was 9.5%. Statistical analyses for amnestic MCI and Alzheimer's disease showed that risk factors for cognitive decline were diabetes mellitus, low activities of daily living, and living alone, and that protective factors were history of exercise and drinking habit.

**Conclusion:**

The present findings suggest that several lifestyle‐related diseases and factors are associated with cognitive decline. These results support similar findings from previous studies and will be helpful for preventing dementia in the future.

## INTRODUCTION

Dementia is a syndrome in which there is loss of memory, orientation, language, problem‐solving, and activities of daily living (ADL). There were over 55 million people living with dementia worldwide in 2021, as estimated by the World Alzheimer Report.^1^ This number of dementia patients is increasing daily and is forecast to reach 78 million in 2030. In Japan, the rate of ageing is steadily increasing, and the number of dementia patients is thus rapidly increasing, which is beginning to attract attention as not only a major medical problem, but also a serious social problem. Therefore, we must take comprehensive measures, including prevention, treatment, and long‐term care, to counteract the increasing number of dementia patients.

Previous studies have proven that lifestyle‐related disease, mental illness, and genetic abnormalities are associated with dementia onset. It is well known from the Rotterdam study that lifestyle‐related diseases cause not only vascular dementia (VaD), but also Alzheimer's disease (AD),^2^ and diabetes mellitus almost doubles the risk of dementia. A meta‐analysis in 2006 suggested that depression may be a risk factor for AD, rather than a prodrome.^3^ The genetic factors of AD have been actively researched, and mutations in the genes such as amyloid precursor protein, presenilin 1, and presenilin 2 are related to early‐onset familial AD.^4^ The functional polymorphisms of apolipoprotein E (APOE) are well known as a strong risk factor for developing AD.^5^

In Japan, many community‐based surveys have been conducted regarding the prevention of dementia. We have investigated the incidence of dementia, rate of behavioural and psychological symptoms of dementia (BPSD), and risk factors and risk of progression from mild cognitive impairment (MCI) to AD by conducting community‐based complete enumeration surveys of dementia in the Nakayama study.^6^, ^7^, ^8^ We reported that diabetes mellitus and a family history of dementia were significant risk factors for progression from MCI to clinically diagnosable AD.^9^ We also reported that the annual conversion rate from MCI to AD was 8.5% per 100 person‐years.^10^

A recent report has demonstrated that modifying 12 risk factors such as less education, hearing loss, and smoking might prevent or delay up to 40% of dementia cases.^11^ However, sufficient explanations have not yet been given for the greatest causes of dementia including AD. We conducted the Nakayama study in 2016 and investigated dementia in Nakayama Town, Ehime Prefecture, Japan. The purpose of this study was to identify risk and protective factors for progression of cognitive impairment by comparing three groups: healthy participants, MCI, and persons with dementia.

## METHODS

### Participants and methods

#### Participants

Nakayama Town in Iyo City is a rural community adjacent to Matsuyama City, the capital of Ehime Prefecture in Japan. This town was selected for its suitable population size for a survey, population stability (few relocations of older people), and active collaboration offered by family doctors.^7^ Past Nakayama surveys were conducted in 1997, 2004, and 2012, and included all residents aged 65 years and older. This survey was conducted from 2016 to 2018, and the participants' basic data and the presence of dementia among them were examined. The survey included all residents aged 65 years and older as in previous Nakayama studies. As of September 1, 2016, the number of participants was 1512. The survey invitation and medical questionnaire were mailed, and all participants underwent magnetic resonance imaging (MRI) using the same device. There were two phases, the first phase (screening interview) and the second phase (clinical evaluation). After the conclusion of the survey, diagnosis and statistical analysis were performed. The survey was conducted using the same protocol in eight regions as the Japan Prospective Studies Collaboration for Ageing and Dementia (JPSC‐AD) study,^12^ and the total number of participants exceeded 10 000. This survey protocol differed from the previous Nakayama studies because it was a multicentre study as part of the JPSC‐AD study. This study was approved by the research ethics committee of Ehime University (Approval Number: 1610004).

#### Methods

##### First phase (screening interview, body measurements, and brain MRI)

First, a briefing for the survey was held at four villages of Nakayama Town. Living condition questionnaires were sent to those who agreed to participate in the survey. The questionnaires included education, work experience, family structure, eating, smoking, drinking, and exercise habits, ADL, instrumental ADL (IADL), sleep quality, and medical history such as lifestyle‐related disease. The Barthel Index,^13^ the Tokyo Metropolitan Institute of Gerontology Index of Competence (TMIG‐IC),^14^ and Pittsburgh Sleep Quality Index (PSQI)^15^ were used for evaluation.

On the day of the survey, the purpose of this study was explained to the participants, and their written, informed consent was obtained. The questionnaires brought by the participants were checked and completed. They underwent examinations such as body measurements, walking function test, blood tests, urinalysis, electrocardiogram, Mini‐Mental State Examination (MMSE), Geriatric Depression Scale (GDS),^16^ and brain MRI. The participants underwent MRI examinations at a medical examination site using a mobile MRI device (Philips 1.5T Achieva).

Detailed interviews about lifestyle habits using questionnaires such as TMIG‐IC, physical and blood examinations, and physiological tests were added in this survey, so that almost all items of the past surveys had been included in the current survey items. Brain imaging was performed in the first phase, which was also different compared to the past surveys (performed in the third phase). The mean length of all examinations was 90 min. To evaluate recent memory in detail, a total of 6 points was scored for recall of MMSE, with each item having a maximum of 2 points. Each item was defined as 2 points for the correct answer without a hint, 1 point for the correct answer with a hint, and 0 points for a mistake. The participants who fulfilled any of the following conditions on the MMSE entered the second phase: (i) total score 26 points or less; (ii) detailed evaluation of recall (2 points for a correct response without a clue and 1 point for a correct response with a clue of each word) was 4 points or less; (iii) incorrect answer of copying; and (iv) suspected cognitive impairment based on behaviour.

##### Second phase (clinical evaluation)

The survey included a detailed medical interview with the caregiver, an original neuropsychological test, and a neurological examination. A neuropsychiatric inventory for evaluating BPSD was also conducted. The neuropsychological evaluation included logical memory of the Wechsler Memory Scale‐Revised, word fluency test, the noise pareidolia test,^17^ and evaluations of other cognitive functions such as orientation, attention, judgement, and general knowledge. Complex neuropsychological examinations differed in each of the past studies. If brain MRI was not available in the first phase, brain computed tomography or MRI at another hospital was evaluated.

#### Diagnosis

Based on the results of the survey, a diagnosis of dementia or MCI was made by several dementia specialists and clinical psychologists. Dementia was diagnosed according to the Diagnostic and Statistical Manual of Mental Disorders, 3rd Revised Edition (DSM‐III‐R).^18^ Petersen's criteria were used for the diagnosis of MCI.^19^ At the same time, dementia participants were classified into subgroups according to the cause of dementia. AD was defined according to the National Institute of Neurological and Communicative Disorders and Stroke‐Alzheimer's Disease and Related Disorders Association criteria (NINCDS‐ADRDA).^20^ VaD was defined according to the National Institute of Neurological Disorders and Stroke‐Association Internationale pour la Recherche et l'Enseignement en Neurosciences (NINDS‐AIREN).^21^ Dementia with Lewy bodies (DLB) was defined according to the Fourth Consensus Report of the DLB Consortium.^22^ Then, a joint meeting was held in the JPSC‐AD^12^ research group, and a final diagnosis was made. The crude prevalence rate was determined by these results, which was then age‐standardised using the World Health Organization World Standard Population (2000–2025) with 5‐year age groups as in a previous paper from this same research program.^23^

#### Statistical analysis

Statistical analysis was performed by dividing the participants into three groups: healthy, amnestic MCI, and AD. AD with cerebrovascular disease (CVD) and VaD were also included. Hypertension, lipid metabolism disorder, diabetes mellitus, drinking habit, smoking habit, exercise habit, obesity, and sleep disturbance were used as the factors related to lifestyle for analysis.

First, comparative analyses of lifestyle‐related diseases and factors were performed among the previously mentioned groups. One‐way analysis of variance (ANOVA) with post hoc Tukey's honestly significant difference (HSD) test was used for statistical analysis of the following factors: age, MMSE, GDS, Barthel Index, TMIG‐IC, blood pressure, blood chemistry results, body mass index (BMI), and PSQI. In addition, a one‐way analysis of covariance (ANCOVA) was performed using age, total Barthel Index score, and total TMIG‐IC score as covariates to account for the effects of age and physical activity level, which were significantly different among the groups. Pearson's Chi‐squared test was used for statistical analysis of the following factors: gender, living alone, and lifestyle habits. Statistical analysis was performed using IBM SPSS version 24.0.

Second, multiple logistic regression analysis was performed among the three groups. Each factor was defined as existing when the participant had any of the following items: (i) hypertension, a history of hypertension, systolic blood pressure ≥ 140 mmHg, diastolic blood pressure ≥ 90 mmHg; (ii) lipid metabolism disorder, a history of lipid metabolism disorder, low‐density lipoprotein cholesterol (LDL‐C) ≥ 140 mg/dL, high‐density lipoprotein cholesterol (HDL‐C) ≤ 39 mg/dL; (iii) diabetes mellitus, history of diabetes mellitus, haemoglobin A1c (HbA1c) ≥ 6.3%, glycated albumin ≥16.6%; (iv) drinking habit, existing drinking habit; (v) smoking habit, existing smoking habit; (vi) exercise habit, existing exercise habit; (vii) obesity, BMI ≥ 25 kg/m^2^; and (viii) sleep disturbance, PSQI ≥6 points. Participants with deficient data were excluded. All analyses were performed using a 95% confidence interval (CI) with IBM SPSS version 24.0. Significance was defined as *P* < 0.05, and descriptive statistics are presented as the means ± standard deviation.

## RESULTS

**Figure 1:**
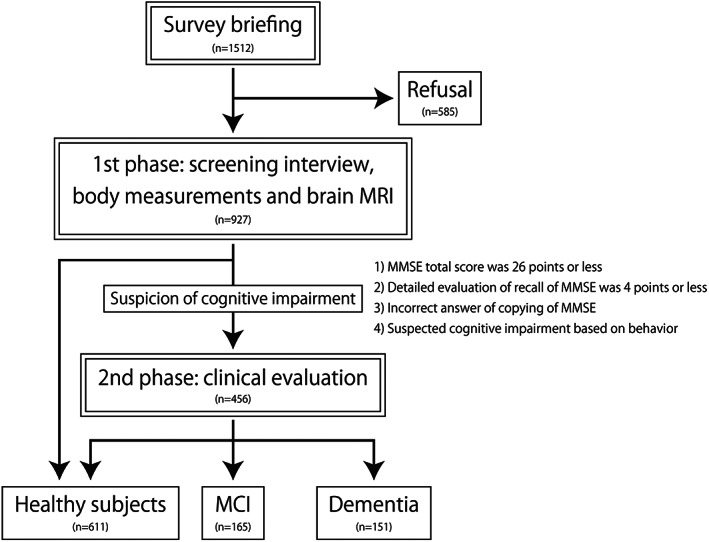
The flow of the baseline survey. After the second phase, participants were diagnosed and divided into healthy, mild cognitive impairment (MCI), and dementia groups. MRI, magnetic resonance imaging. MMSE, Mini‐Mental State Examination.

**Figure 2:**
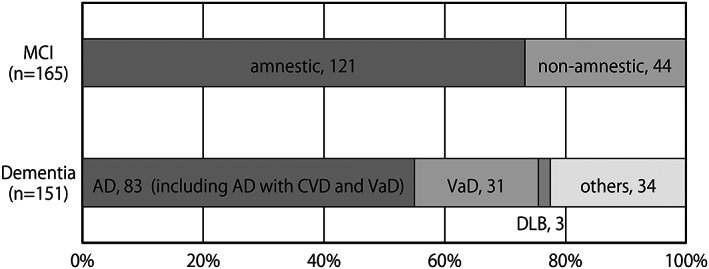
The breakdown of mild cognitive impairment (MCI) and dementia. AD, Alzheimer's disease. CVD, cerebrovascular disease. VaD, vascular dementia. DLB, dementia with Lewy bodies.

The flow of the survey and the number of persons at each phase are shown in Fig. 1. Of the 1512 residents aged 65 years and older on the reference date, 927 participants (61.3%) participated in this survey. There were 585 non‐participants; 335 did not reply or could not be contacted, 100 refused to participate, 88 died before the survey, 50 entered a hospital or a facility for the aged, four moved to another town, and eight were categorised as other (unknown). The diagnostic results of the participants are shown in Fig. 2. At the baseline survey, of the participants, 611 (65.9%) were healthy, 165 (17.8%) had MCI, and 151 (16.3%) had dementia. The age‐standardised prevalence of dementia in people aged 65 years or older using the World Health Organization World Standard Population was 9.5%. Of those with MCI, 121 had amnestic MCI, and 44 had non‐amnestic MCI. Of those with dementia, 83 were diagnosed with AD (19 were AD with VaD; core conditions were AD), 31 were diagnosed with VaD, three were diagnosed with DLB, and 34 were diagnosed with dementia caused by other pathology.

Table 1 shows the demographic data of three groups and results of one‐way ANOVA and Pearson's Chi‐squared test: healthy, amnestic MCI, and AD. Three participants (MCI: one participant, AD: two participants) without enough data were excluded. There were missing data in some items (GDS (AD: four participants), blood pressure (healthy: one participant), drinking habit (healthy: two participants, AD: 12 participants), smoking habit (healthy: two participants, AD: 12 participants), and BMI (healthy: one participant, AD: three participants)). The mean values and percentages were calculated by removing missing data.

**Table 1:** Baseline characteristics of healthy, amnestic MCI, and AD participants

	Healthy (*n* = 611)		Amnestic MCI (*n* = 120)		AD (*n* = 81)		*P*‐value
	Mean (SD)	*n* (%)	Mean (SD)	*n* (%)	Mean (SD)	*n* (%)	
Demographics							
Age, years^†^	74.5 (6.9)		80.4 (6.5)		85.9 (5.7)		<0.001^§^
Female^‡^		348 (57.0%)		67 (55.8%)		56 (69.1%)	0.099
MMSE total score^†^	28.5 (1.8)		25.1 (1.8)		15.8 (6.5)		<0.001^¶^
GDS total score^†^	2.3 (2.3)		2.6 (2.1)		2.8 (2.7)		0.153
Barthel Index total score^†^	98.8 (5.2)		97.5 (6.1)		80.9 (28.1)		<0.001^††^
TMIG‐IC total score^†^	11.9 (2.0)		10.9 (2.8)		4.6 (4.8)		<0.001^¶^
Living alone^‡^		112 (18.3%)		31 (25.8%)		9 (11.1%)	0.028*
Lifestyle‐related disease							
History of hypertension^‡^		370 (60.6%)		86 (71.7%)		52 (64.2%)	0.068
Systolic blood pressure, mmHg^†^	138.6 (16.0)		136.2 (14.9)		135.7 (20.2)		0.292
Diastolic blood pressure, mmHg^†^	77.1 (11.1)		75.4 (9.9)		74.9 (9.7)		0.193
History of lipid metabolism disorder^‡^		223 (36.5%)		42 (35.0%)		21 (25.9%)	0.173
LDL‐cholesterol, mg/dL^†^	111.5 (27.1)		108.4 (25.8)		107.0 (25.9)		0.343
HDL‐cholesterol, mg/dL^†^	59.7 (16.8)		56.0 (14.4)		56.3 (15.2)		0.062
History of diabetes mellitus^‡^		80 (13.1%)		11 (9.2%)		11 (13.6%)	0.474
HbA1c, %^†^	5.8 (0.8)		5.8 (0.6)		5.9 (0.8)		0.566
Glycated albumin, %^†^	15.7 (3.2)		15.9 (2.2)		17.4 (3.8)		<0.005^‡‡^
Glycated albumin/HbA1c ratio^†^	2.70 (0.30)		2.75 (0.28)		2.93 (0.35)		<0.001^‡‡^
Lifestyle‐related factors							
History of drinking^‡^		265 (43.5%)		45 (37.5%)		17 (28.3%)	0.007**
History of smoking^‡^		166 (27.3%)		39 (32.5%)		13 (18.8%)	0.128
Exercise habit^‡^		311 (50.9%)		73 (60.8%)		31 (38.3%)	0.007**
BMI^†^	23.5 (3.4)		23.5 (3.2)		23.1 (3.5)		0.617
PSQI total score^†^	4.4 (3.4)		4.8 (3.8)		3.9 (3.1)		0.171

Abbreviations: MCI, mild cognitive impairment; AD, Alzheimer's disease; SD, standard deviation; MMSE, Mini‐Mental State Examination; GDS, Geriatric Depression Scale; TMIG‐IC, Tokyo Metropolitan Institute of Gerontology Index of Competence; LDL‐C, low‐density lipoprotein cholesterol; HDL‐C, high‐density lipoprotein cholesterol; HbA1c, haemoglobin A1c; BMI, body mass index; PSQI, Pittsburgh Sleep Quality Index.

^†^ One‐way analysis of variance with the post hoc Tukey's honestly significant difference test.

^‡^ Pearson's Chi‐squared test.

^§^ Healthy < amnestic MCI < AD.

^¶^ AD < amnestic MCI < Healthy.

^††^ AD< Healthy, AD < amnestic MCI.

^‡‡^ Healthy < AD, amnestic MCI < AD.

* *P* < 0.05;

** *P* < 0.01.

On one‐way ANOVA with Tukey's post hoc HSD test and Pearson's Chi‐squared test comparing among the three groups, significant differences appeared in some factors. Age was significantly higher in amnestic MCI than in healthy participants, and higher in AD than in amnestic MCI (*P* < 0.001). MMSE and TMIG‐IC total scores were significantly lower in amnestic MCI than in healthy participants, and lower in AD than in amnestic MCI (both *P*‐values <0.001). Glycated albumin and the glycated albumin/HbA1c ratio were significantly higher in AD than in healthy participants and amnestic MCI (*P* < 0.005 and <0.001). The Barthel Index total score was significantly lower in AD than in both healthy participants and amnestic MCI (*P* < 0.001). Statistical analysis by one‐way ANCOVA adjusted for age and physical activity revealed that the significant differences in glycated albumin and glycated albumin/HbA1c ratio disappeared, but the PSQI total score was significantly lower in AD (*P*‐value <0.001). The results for the other factors were similar to those of the ANOVA. The proportion living alone was significantly higher in amnestic MCI (*P* = 0.028), and the proportions of a history of drinking and exercise habit were significantly lower in AD (both *P*‐values 0.007).

The results of multiple logistic regression analysis are shown in Table 2. The statistical analyses were performed by excluding 20 participants (five healthy and 15 AD) with missing data. There were no significant differences between healthy participants and amnestic MCI participants. Logistic regression model results showed that AD had a higher proportion of diabetes mellitus than healthy participants (odds ratio = 1.92; 95% CI 1.04, 3.54) and amnestic MCI (odds ratio = 2.36; 95% CI 1.18, 4.74).

**Table 2:** Comparison among three groups of lifestyle‐related factors on multiple logistic regression analysis

	AD vs. Healthy		Amnestic MCI vs. Healthy		AD vs. Amnestic MCI	
	*P*‐value	Odds ratio [95% CI]	*P*‐value	Odds ratio [95% CI]	*P*‐value	Odds ratio [95% CI]
Hypertension	0.181	0.60 [0.29, 1.27]	0.943	1.02 [0.59, 1.75]	0.466	0.73 [0.31, 1.71]
Lipid metabolism disorder	0.651	0.87 [0.47, 1.61]	0.372	0.82 [0.54, 1.26]	0.513	0.79 [0.38, 1.61]
Diabetes mellitus	0.037*	1.92 [1.04, 3.54]	0.837	0.95 [0.60, 1.52]	0.016*	2.36 [1.18, 4.74]
Drinking habit	0.223	0.59 [0.25, 1.38]	0.205	0.69 [0.38, 1.23]	0.800	1.13 [0.45, 2.81]
Smoking habit	0.637	1.28 [0.45, 3.63]	0.132	1.67 [0.86, 3.23]	0.094	0.37 [0.12, 1.18]
Exercise habit	0.549	0.83 [0.45, 1.53]	0.071	1.49 [0.97, 2.29]	0.107	0.56 [0.28, 1.13]
Obesity	0.912	0.96 [0.49, 1.89]	0.600	1.13 [0.72, 1.77]	0.802	0.91 [0.42, 1.94]
Sleep disturbance	0.275	0.69 [0.35, 1.35]	0.561	1.14 [0.73, 1.79]	0.970	0.99 [0.45, 2.15]
Age	<0.001**	0.80 [0.76, 0.84]	<0.001**	0.89 [0.86, 0.92]	<0.001**	0.88 [0.82, 0.93]
Gender	0.578	1.31 [0.51, 3.34]	0.784	0.91 [0.45, 1.83]	0.661	0.78 [0.26, 2.34]

Abbreviations: MCI, mild cognitive impairment; AD, Alzheimer's disease.

* *P* < 0.05;

** *P* < 0.001.

## DISCUSSION

A community‐based complete enumeration study about dementia was performed in Nakayama Town in Iyo City, Ehime Prefecture, Japan, and the recent prevalence and risk factors for dementia were identified. There are few extensive investigations similar to the present study. It is widely known that diabetes mellitus is a risk factor for AD, and this was confirmed by the present enumeration study. In addition, it suggests that other risk factors are low ADL and living alone, and protective factors for cognitive function are a history of exercise and drinking habits.

We have been conducting four complete enumeration surveys in the same region since 1997. The crude and age‐standardised prevalence rates of dementia in the present study were 16.3% and 9.5%, respectively, significantly higher than in the first survey (4.9% and 4.5%, respectively) in 1997.^8^, ^23^ One of the reasons for the high prevalence was the increase in the average life expectancy and the ageing population in Japan. However, the prevalence has been increasing compared to the ageing population due to not only ageing, but also other factors. We suggest that the rising prevalence of AD might be related to changes in lifestyle or nutrition.^23^ The previous Nakayama survey found that diabetes mellitus and a family history of dementia were positively associated with progression to AD.^8^ We have not investigated family history, but we derived similar results for diabetes mellitus being a risk factor for AD. Therefore, we focused on diabetes mellitus and in addition noted other possible factors.

A recent meta‐analysis also suggested that persons with diabetes had a higher risk (relative risk 1.6) of AD compared to those without diabetes.^24^ After the onset of dementia, the change in diet and motor functional decline may lead to diabetes, but there are few reports on dementia itself being a risk factor for diabetes. On the other hand, much evidence has indicated that diabetes was a risk factor for dementia, and the result of the present study was similar. It is considered that the risk of dementia increases through a variety of mechanisms in diabetes mellitus. The risk factors for cognitive dysfunction in diabetes mellitus are divided into vascular and metabolic factors.^25^ Diabetes mellitus increases CVD, as well as hypertension, and decreases cognitive function.^26^ Hypertension is a risk factor for the development of AD through CVD, and the renin‐angiotensin system has been implicated in AD.^27^, ^28^ Angiotensin II via the angiotensin II type 1 receptor is reported to increase brain amyloid‐β levels by increasing amyloid precursor protein messenger RNA, β‐secretase activity, and presenilin expression,^29^ which is considered to be the basis for the vascular risk factors for developing AD. Metabolic factors of AD development are thought to include amyloid‐β, tau phosphorylation, glycogen‐synthasekinase‐3β (GSK‐3β), and oxidative stress‐mediated mechanisms. Increased oxidative stress and insulin resistance with diabetes mellitus reportedly cause the accumulation of amyloid‐peptide and neurofibrillary tangles in the brain.^30^, ^31^ Some research in recent years has focused on GSK‐3β related to both AD and diabetes mellitus.^32^ GSK‐3β is an enzyme implicated in the phosphorylation of various proteins, and overactivity of GSK‐3β causes a failure to convert glucose to glycogen, which results in hyperglycaemia and diabetes mellitus. Recent studies have reported that the glycated albumin/HbA1c ratio was associated with the development of AD,^33^ and it was also significantly elevated in the AD group of the present study. The exact mechanisms by which AD increased with a higher glycated albumin/HbA1c ratio are not known. Glycated albumin has been considered to reflect postprandial hyperglycaemia and glucose excursion, and postprandial hyperglycaemia was significantly associated with an increased risk of AD in virtue of oxidative stress. These results suggest that diabetes mellitus increased the risk of AD, and the present study showed similar results to previous studies and supports this theory.

Other factors besides diabetes mellitus were apathy, social isolation, and drinking habit. Apathy was the most frequent BPSD and the symptom with frequencies exceeding 50% in AD patients.^34^ It is known that the worsening of apathy can lead to inadequate exercise habits and decreased ADL. In a meta‐analysis of prospective studies, it was found that participants who reported performing a high level of activity had a 38% reduced risk of cognitive decline with respect to those who reported being sedentary, and even low‐to‐moderate levels of physical activity had a preventive effect on cognitive decline.^35^ In another meta‐analysis, the relative risk of AD in the highest physical activity category compared with the lowest was 0.55.^36^ There were some reports that exercise reduces beta‐amyloid deposition and prevents amyloid‐β‐associated neurotoxicity in the brain.^37^, ^38^, ^39^ AD participants had low ADL and lack of exercise habits in the present study, and it is suggested that these factors and dementia are related to each other, as in previous studies.

The number of older people living alone who are at high risk of social isolation is increasing with advancement of the ageing society, and opportunities for social activities and communication are being lost. A recent study demonstrated that social isolation was associated with poor cognitive function.^40^ In addition, it was reported that people with depression or anxiety experience higher levels of social isolation than people without them,^41^ but there was no difference among the three groups in the present study. The biological mechanism that causes cognitive decline by living alone is unclear. It is possible that decreased activity and communication by living alone are related to cognitive dysfunction. In the present study, there was a higher rate of solitary living in the amnestic MCI group and a lower rate in the AD group. The present data suggest that social isolation may reduce social activity and communication and cause cognitive impairment. As the cognitive impairment progressed further, patients were more likely to be admitted to a nursing home, which might have reduced the rate of living alone in AD.

Previous studies have shown that drinking affects cognitive function, having both protective and harmful or destructive effects. It has been reported that small amounts of alcohol might be protective against AD compared with abstention.^42^, ^43^ It was considered that the protective effects of alcohol are related to cardiovascular mechanisms. In this study, the rate of drinking habits in healthy participants was higher than in amnestic MCI and AD participants. As mentioned previously, it is possible that drinking habit itself has a protective effect on cognitive function, or social activity decline leads to fewer opportunities to drink as cognitive impairment progresses. It is assumed that social activities, as well as drinking, are associated with preventing decline in cognitive function because some districts in this survey area hold a regular meeting with drinking. On the other hand, it is widely known that heavy alcohol consumption causes Wernicke‐Korsakoff's syndrome and brain damage, and recent studies found that alcohol consumption did not protect cognitive function.^44^, ^45^ The meta‐analysis we conducted also showed that a functional genetic allele of ALDH2, which reduces alcohol metabolism, is a risk factor for AD in Asians.^46^ Therefore, the relationship between alcohol and cognitive function needs to be carefully examined, and we do not strongly recommend drinking alcoholic beverages by any means. ANCOVA adjusted for age and physical activity found that sleep disturbance (PSQI) was lower in AD patients, probably reflecting the lack of symptom awareness in AD patients because the PSQI is self‐administered.

There are several limitations to this study. The participation rate was somewhat low despite mailing the survey invitation and calling the participants several times. The rate was likely low due to the time required for detailed examinations including MRI. However, participants were unbiased because the survey was conducted not only at the venue, but also door‐to‐door. The statistical analysis included AD participants with complications of VaD, which had to be included due to the small number of participants. Core conditions of AD participants in the present survey were not VaD but AD, and several previous studies have treated AD with CVD as AD. Therefore, the selection of all AD participants was appropriate. The statistical analyses in the present study targeted lifestyle‐related diseases and factors and did not examine other factors such as genetic factors and other physical diseases. The number of participants was not large enough to conduct analyses including all factors. Accordingly, we plan to analyze other factors separately and conduct more precise examinations in the future. In addition, we will reveal significant relationships by longitudinal cohort process.

## CONCLUSION

The present data indicate that diabetes mellitus is a risk factor for AD. In addition, several other lifestyle‐related diseases and factors were found to be associated with cognitive function, and some may lead to prevention of AD. These results support the factors identified in the past studies. A follow‐up study is currently underway to precisely identify the risk and protective factors of cognitive decline.

## Disclosure

The authors declare they have no conflicts of interest in the research.

## Data Availability

The data that support the findings of this study are available on request from the corresponding author. The data are not publicly available due to privacy or ethical restrictions.
